# Hybrid Materials: A Metareview

**DOI:** 10.1021/acs.chemmater.3c01878

**Published:** 2023-12-18

**Authors:** Pedro Gomez-Romero, Anukriti Pokhriyal, Daniel Rueda-García, Leandro N. Bengoa, Rosa M. González-Gil

**Affiliations:** † Novel Energy-Oriented Materials Group at Catalan Institute of Nanoscience and Nanotechnology (ICN2) CSIC and BIST, Campus UAB, Bellaterra, 08193 Barcelona, Spain; ‡ Napptilus Battery Labs, Tech Barcelona 01, Plaça de Pau Vila, 1, Oficina 2B, 08039 Barcelona, Spain

## Abstract

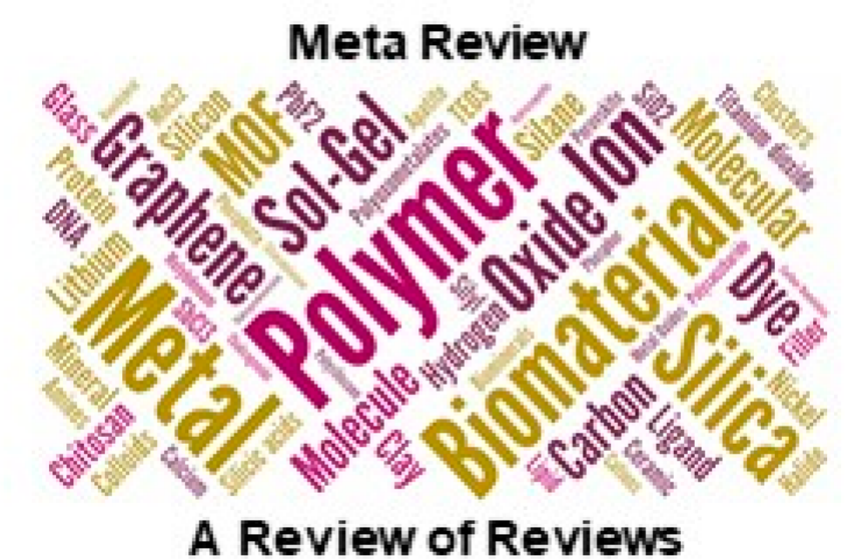

The field of hybrid materials has grown so wildly in the last 30 years that writing a comprehensive review has turned into an impossible mission. Yet, the need for a general view of the field remains, and it would be certainly useful to draw a scientific and technological map connecting the dots of the very different subfields of hybrid materials, a map which could relate the essential common characteristics of these fascinating materials while providing an overview of the very different combinations, synthetic approaches, and final applications formulated in this field, which has become a whole world. That is why we decided to write this metareview, that is, a review of reviews that could provide an eagle’s eye view of a complex and varied landscape of materials which nevertheless share a common driving force: the power of hybridization.

## 1 Introduction: From a Tree to a Meadow

Hybrid materials bring selected organic and inorganic compounds into novel materials that combine their best properties, resulting in synergic combinations with unique properties and improved performance in a wide variety of applications. The field is so broad that it has been the subject of an ever-growing number of reviews and books.^1−5^ But this multiplicity has led to a sustained divergence and fragmentation of the field. Books on hybrids keep being published, but they tend to be more and more focused on particular types or applications of hybrids. This is perfect for specialized practitioners but leaves a gap to fill for young researchers in search of inspiration. This *metareview* aspires to fill that gap by bringing selected reviews and feature articles into a coherent comprehensible article that could convey the awesome nature but also the many opportunities to contribute to this field.

**Figure 1:**
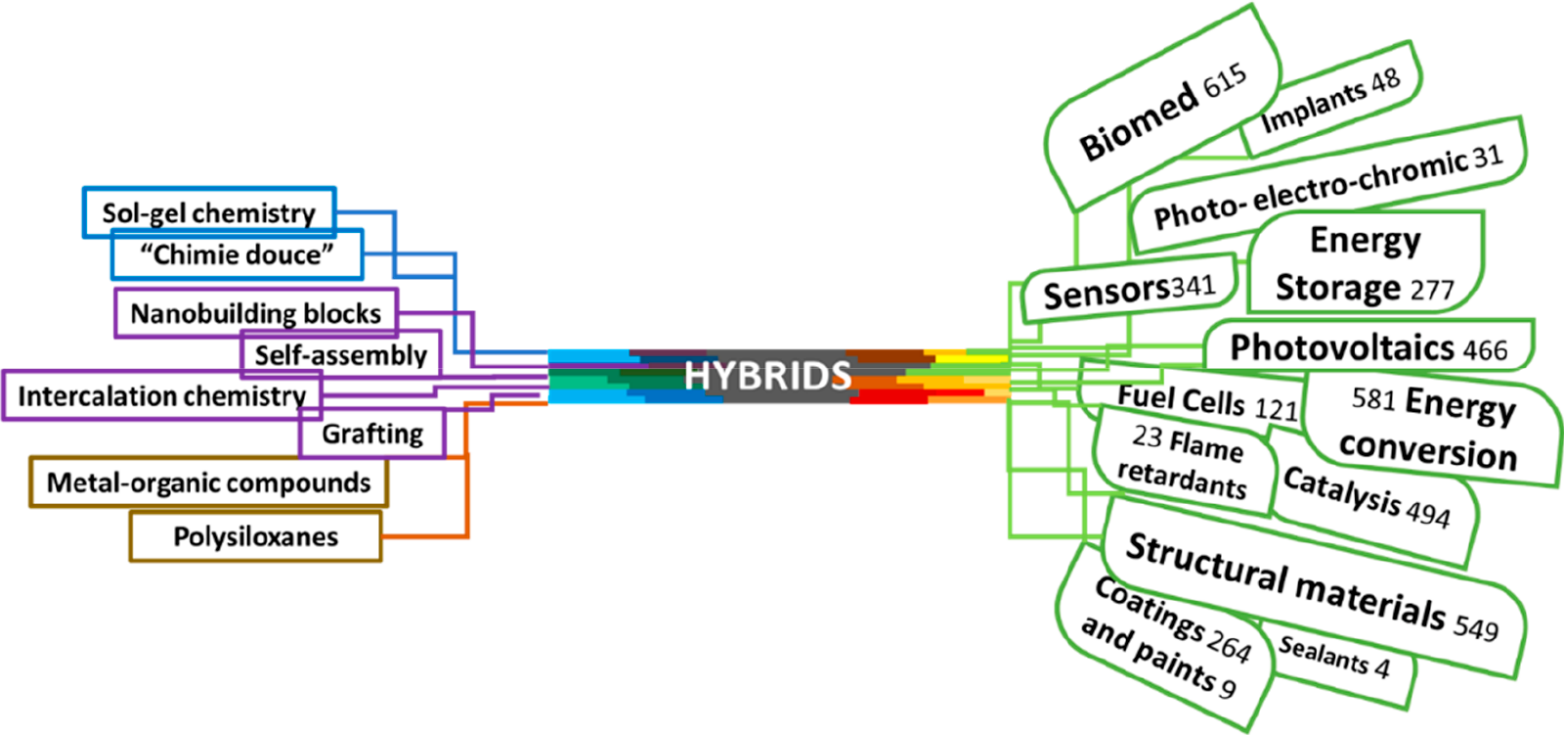
Graphical representation of hybrid materials: from their chemical roots to the wide variety of final applications. The numbers indicated for each application correspond to the number or reviews found for “hybrid materials” AND the corresponding application (Web of Science). These figures are intended as a mere estimate of the relative abundance of specialized reviews, but they are not mutually exclusive (i.e., a review on “implants” could also be included in “biomed”).

The field of hybrid materials could be considered a tree. A classical metaphoric image of a tree of knowledge, which immediately evokes a structure of roots stem and branches (Figure 1).

The visible branches of this tree have led to a wide variety of final applications that are so varied and different and will be discussed later in detail. The roots of the field of hybrid materials are multiple, as the tree image itself properly portrays. The multiplicity of roots will probably lead to unforgivable omissions if we could dare try to make a comprehensive list of all of the scientists contributing to each of these foundations. That is why we will refer the reader to our first reviewed review, a great account on the history of hybrid materials by Faustini, Nicole, Ruiz-Hitzky, and Sanchez,^2^ a review that blends nature, art, science, and technology in a very inspiring way. But this metareview is not just a compendium of the best reviews in the field. Databases and artificial intelligence are smart enough these days to do that task on their own. That is why it is our privilege to share some insights, some human insights, related to the origins.

Thus, why would anyone try to make a material with the best properties of both glass and polymers?

The answer is simple: to get a better material, transparent, lightweight but resistant, hard but not brittle, and manufacturable at low temperatures but able to stand elevated temperatures. You name it.

And why would anyone try to make a compound involving organic and inorganic chemistry, risking being left out of both organic and inorganic departments and laboratories? Intellectual audacity could have something to do with it.

This dual path of fundamental and applied driving forces acting in parallel or in series is very characteristic of the hybrid endeavor. For example, the synthesis of poly siloxanes in the beginning of the 20th century, represented the fundamental development of silicones.^2^ Forty years later, practical efforts followed to put together the best properties of inorganic quartz and organic polymers in a new type of synthetic rubber for the sake of national security. Indeed, in January 1945, the USA was still at war with Japan, a circumstance that showed clearly on the cover of the January issue of *Popular Science* magazine, featuring a demonized kamikaze pilot about to attack the reader. Inside, among many other war-related technology breakthroughs, there was an article titled “Here is putty with a bounce.” Silly putty was an unexpected byproduct of the race to convert polysiloxanes from useless compounds into useful silicone materials.^6^ But the use of silicones in non-Newtonian fluid toys or in improved gaskets or sealants was just the beginning. The chemistry of poly siloxanes grew and evolved in parallel to their applications, and more and more complex varieties, like poly silsexquioxanes, were added to the family^7−11^ in a growing trend that has not stopped since then.

The path from intercalation chemistry to hybrid materials is another major example of a field with consolidated fundamental knowledge breaking its own boundaries to grow into a new field within materials science. Thus, the use of layered inorganic phases (typically intercalating simple metal cations) as hosts for the intercalation of complex organic cations and polycations marked the starting point for an explosion of a new type of hybrid chemistry.^12−15^ The new knowledge and the new tools developed along the way led to the discovery of early hybrid materials in unexpected ancient materials such as Maya blue or thin Chinese porcelain. Most importantly, this new knowledge and synthetic and analytical tools led to the blooming of hybrid materials rooted in the intercalation chemistry of silicates, oxides, or other chalcogenides, as well as new layered phases.^2,12,16−18^ In addition, they expanded from cationic to anionic intercalates, as well as neutral and solvent and solvated species.

Sol–gel chemistry constitutes another primary root in the early development of hybrid materials. The French school transitioning from “Chimie du Solide” to “Chimie Douce”^19^ provided a solid community of bright scientists setting the basis for the synthesis of new metastable and complex hybrid phases, as well as pioneering sustainable, low-temperature routes to ceramics and hybrid materials.

Clément Sanchez played a seminal role in all these fundamental fields and led their consolidation into the broader field of hybrid materials.^1,2,5,12,20−23^ New approaches were added to the discipline, from new types of materials to new synthetic methods, leading to an unprecedented variety of fields in the field. New materials like those formed by the simple ionic integration of polyanion and/or polycation polymers with charged complexes or clusters^17,24−26^ or the whole emerging field of metal–organic frameworks (MOFs) are hybrid by their own definition.^27−30^ There are also new synthetic approaches, like the use of preformed nanobuilding blocks for the construction of sophisticated hierarchically structured materials.^12,22,29,31−34^

Variety led to the need for classifications to add some orderto the profusion of materials. Type I and type II hybrids and organic–inorganic as well as inorganic–organic hybrid materials were proposed, as will be discussed below. However, far more important than these classifications is an understanding of the factors common to all hybrid materials.

Underlying the search for hybrid materials, we will always find the desire to take advantage of the best properties of each of the components, leading in the most favorable cases to synergic properties that go beyond the simple addition of the component’s properties. This ***synergy*** has been exemplified and reviewed for diverse types of hybrids^35^ and is one of the factors common to hybrid materials when we look at the final outcome and resulting properties.

But if we consider the chemical genesis of hybrid materials, we will also find very interesting recurring characteristics which are suggestive of general strategies that can be followed for the synthesis of new hybrids. Thus, the concept of self-assembly is present in a wide range of hybrid materials synthesis,^12,13,36−42^ from spontaneous sol–gel processes to the designed assembly of nano building blocks or the crystal growth of metal–organic frameworks. Whether it is revealed by discovery as in sol–gel growth or realized by design as in the case of MOFs, self-assembly is at the heart of the bottom-up growth of hybrid materials. And yet, another large group of hybrid materials are made through bond-engineering, that is, through the purposeful creation of covalent bonds between the dissimilar components to be combined in the hybrid. This approach, which has been dubbed “grafting”, represents a radically different approach to spontaneous self-assembly and takes us naturally to the discussion of the wide variety of hybrid materials, their classification, and an attempt to put some Cartesian order in the methods used to synthesize them. Or should we say grow them? Because after this brief introduction, it is already clear that the field of hybrid materials has turned into a complex landscape with a rich orography. The tree of hybrids has turned into a meadow with many trees.

## 2 Hybrid Materials: A Land of Multidisciplinarity

“A land of multidisciplinarity” was precisely the expression used by Judeinstein and Sanchez in a seminal 1996 paper to describe the emerging field of hybrid materials.^20^ They appeared as an elegant way to get new materials with multifaceted and tailored features from the combination of organic and inorganic phases at the micro-, meso-, and nanoscale levels.

**Figure 2:**
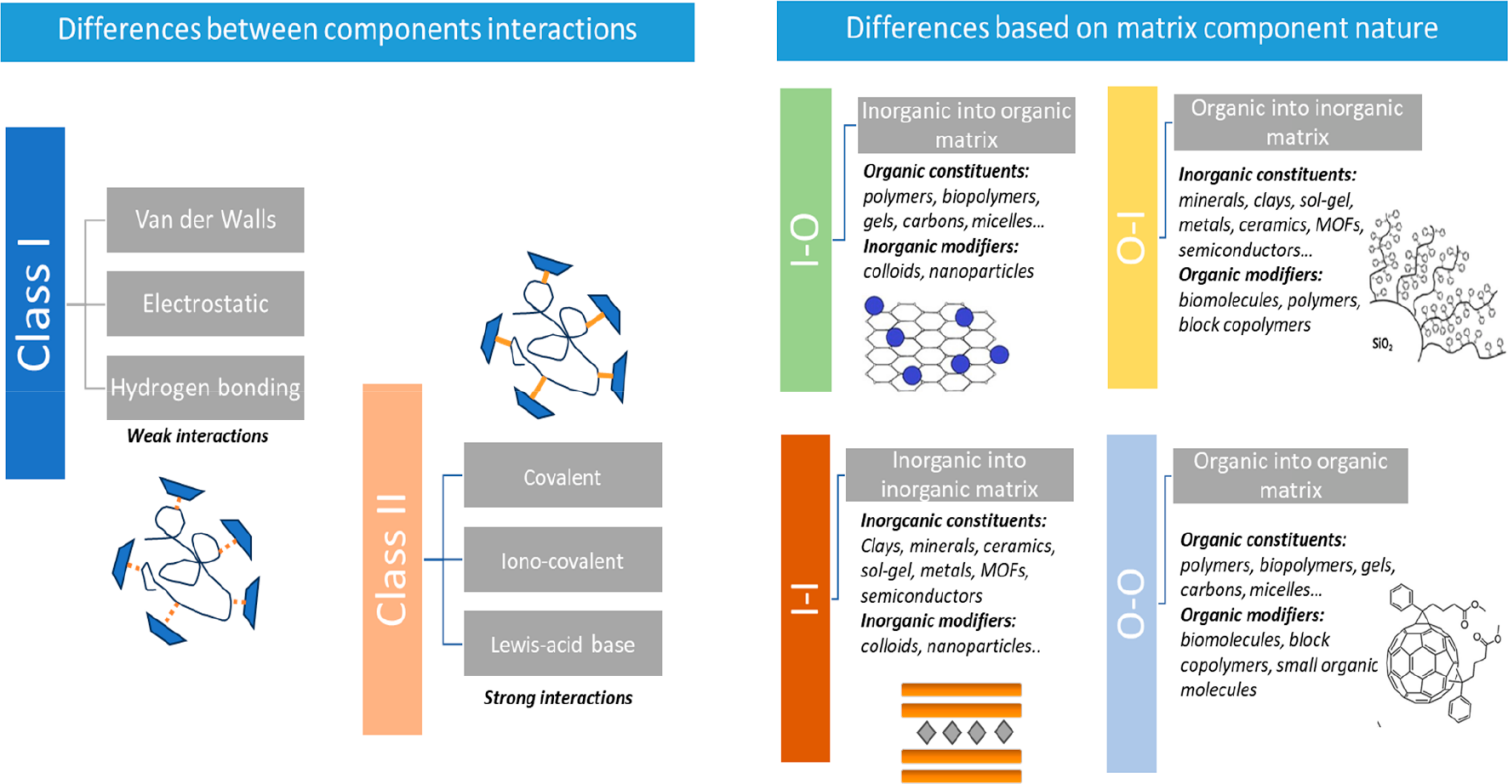
Hybrid materials are general classifications. On the left, classification based on the differences between the interactions of the components, having class I and class II hybrid materials. On the right, the classification according to the matrix and filler component nature, being divided into four classifications: I–O, O–I, I–I, and O–O types.

Two types of classification are commonly used to sort hybrid materials in the literature (Figure 2). The first and most prevalent one is based on the type of interactions between the organic and inorganic components, whereas the second considers which component acts as the dominant matrix and which one acts as guest.

The first classification, originally proposed by Judeinstein and Sanchez,^20^ considers Class I hybrids as those formed through weak interactions between the organic and inorganic components. This includes hydrogen bonding, electrostatic, and/or van der Waals interactions. On the other hand, class II hybrids are based on strong chemical interactions such as covalent bonds. However, this classification sometimes is ambiguous because, in the same hybrid material, strong and weak interactions can coexist.

Class I hybrid materials are commonly prepared by sol–gel processes, self-assembly, and in situ polymerization methods. In this sense, sol–gel synthesis, developed in 1930s,^43^ has become one of the major research lines in the broad field of hybrid materials synthesis.^22,31,43−57^ Organic molecules or monomers embedded in sol–gel matrices are common examples that could present a large diversity in their structures and final properties leading to many multifunctional materials. Polymers filled with inorganic clusters, organogels, and biological-based hybrid materials are other extended examples of class I hybrids.^7,13,24,40,43,45,51−53,58−60^ In the case of Class II hybrid materials, covalent or ion-covalent bonds are present between the organic and inorganic phases.^20,46^ In this sense, the grafting methodology, appears as a common strategy to form class II hybrid materials.^13,14,43,44,53,58,61−64^ This method, sometimes applied as a postsynthetic step, normally implies the attachment of functional organic molecules on the surface of inorganic moieties (type I–O), such as silica, titania, other metal oxides, and/or carbon surfaces.^3,23,65,66^ Sol–gel is again one of the most used as a suitable methodology for the preparation of this class of materials, with the development of hybrid materials from polyfunctional alkoxysilanes a typical example for obtaining a wide range of functional materials due to their high versatility.^67−69^ However, electrochemical grafting using aryl diazonium salts is the most used in the case of carbonaceous matrices. This method is based on the electrochemical reduction of diazonium salts, which decompose into radicals and nitrogen gas, giving a direct C–C bond.^64,70^ Other typical methods commonly used to prepare type II hybrid materials are self-assembly synthesis,^13,36,41^ template-assisted synthesis,^24,63,71^ hydrothermal,^12,57,72,73^ or layer-by-layer deposition.^49,74,75^

**Figure 3:**
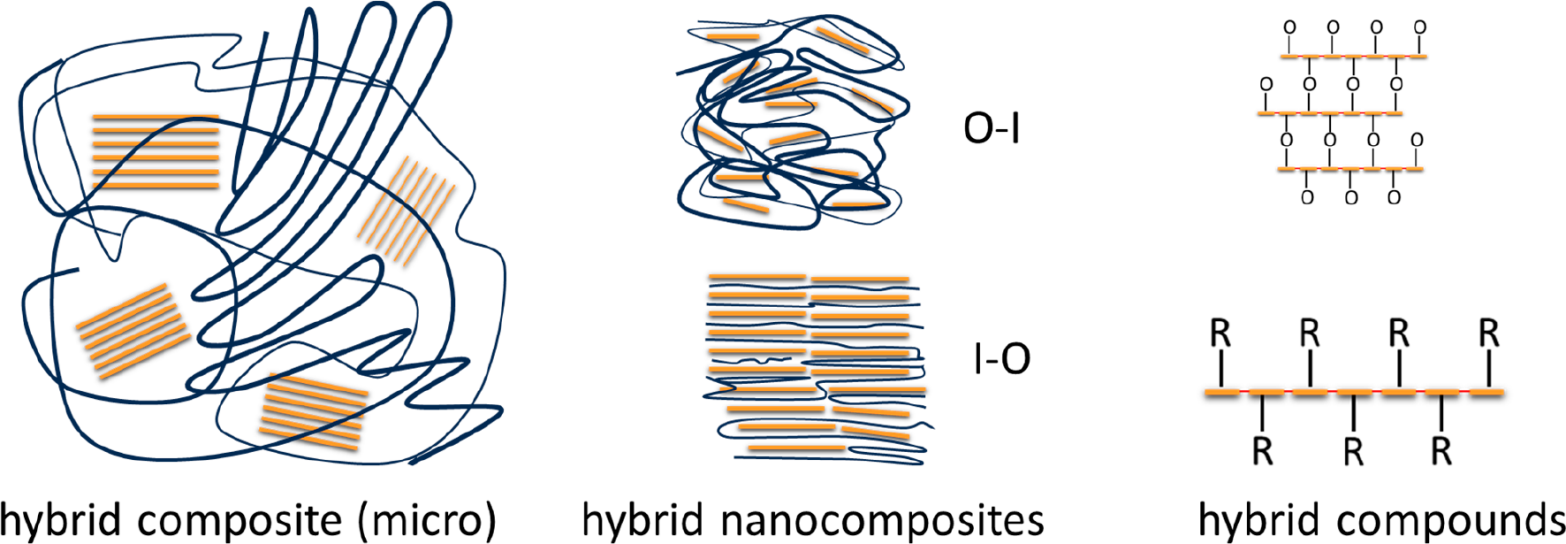
Schematic representation of different types of hybrid materials where the dispersion of organic and inorganic components takes place at various levels. Hybrid composites (left) are not mere physical mixtures, since the interface between organic (black lines denoting a polymer) and inorganic (orange lines representing a layered phase or aggregated rods) has an enhanced relevance. Nevertheless, in these cases, domains of the individual components remain. In a hybrid nanocomposite (middle), those domains are blurred or vanished. It is in this particular case that Organic–Inorganic or Inorganic–Organic materials could be distinguished depending on the matrix dominating the structure. Finally in hybrid compound materials like polysiloxanes or MOFs (right), organic and inorganic moieties are orderly bonded at the molecular level.

The second criterion for the classification of hybrid materials focuses on the nature of the dominating structural matrix component and the one that is hosted. According to this classification, hybrid materials can be divided into two main groups: organic–inorganic (OI), when the matrix is an organic phase, and inorganic–organic (IO) hybrids, when there is an inorganic host where organic guests are integrated.^25^ It should be noted that in a broad sense, combinations of dissimilar inorganics could also be considered as hybrid materials, which in that case could be classified as inorganic–inorganic hybrids (II), for example inorganic complexes, clusters, or nanoparticles intercalated in mineral phases, like layered silicates,^12,16^ and a similar situation could be considered for the existence of all-organic (O–O) hybrids.^76,77^ This classification is most significant in the case of nanocomposites (Figure 3), where one of the two components dominates the structures.

At this point we should note that the terms *composite* or *nanocomposite* are frequently used for describing combinations of dissimilar phases to create new materials.^78^ In this respect, nanocomposites and hybrid materials could seem to be synonym terms. They respond, however, to different classification criteria: hybrids refer to the combination of dissimilar components, normally organic and inorganic, whereas composites and nanocomposites refer to their degree of dispersion. Figure 3 could shed some light on this issue. A hybrid composite material combines organic and inorganic phases beyond a simple physical mixture but maintains the integrity of each of the phases, and thus, the material keeps domains of each. A hybrid nanocomposite pushes further the degree of dispersion (for example, by delamination of layered phases or disaggregation of linear polymeric chains, eventually eliminating the presence of domains of the individual components. At this stage of integration, the classification as the OI or IO mentioned above is fully meaningful. Finally, hybrid compounds such as polysiloxanes or like metal–organic frameworks (MOFs) represent the ultimate dispersion of organic and inorganic moieties, which are orderly bonded at the molecular level.

Setting aside the issue of classifications, it is very enlightening to analyze the composition of hybrid materials and their structures, two important aspects which are frequently correlated.

**Figure 4:**
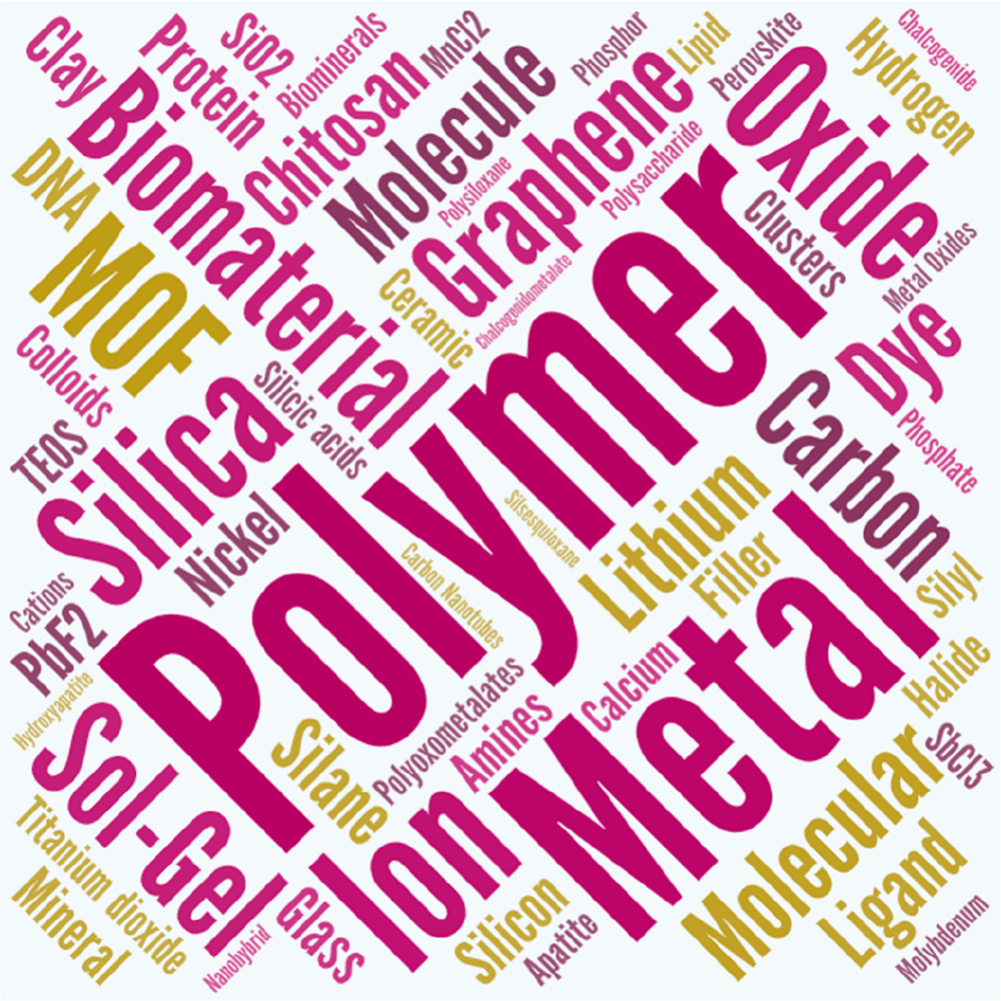
Word cloud generated from the text contained in the abstracts of reviews referenced in this metareview. Only terms related to the composition were kept. This image provides a semiquantitative picture of the relative importance of several types of materials and chemicals in the preparation of the hybrid materials referenced. It should not be taken as a statistical estimate given the relatively reduced size of the sampled text.

Concerning composition and faced with the overwhelming variety of materials and type of materials used to prepare hybrids, we have carried out a word-cloud exercise by graphically picturing the words referring to chemical components (specific or generic) with fonts of size proportional to the appearance of those terms in the abstracts of the reviews referenced in this article. Of course, the result, which is depicted in Figure 4, is not intended to convey comprehensive or quantitative conclusions. Instead, it tries to give a feeling of the types of materials most frequently mentioned in our references as substantial components of hybrid materials. It is remarkable, although not too surprising, the preeminence of polymers, silica/SiO_2_, biomaterials, and metals (including “transition metals”). But it is also noteworthy the emergence of terms like MOFs (metal–organic frameworks) or perovskites.

**Figure 5:**
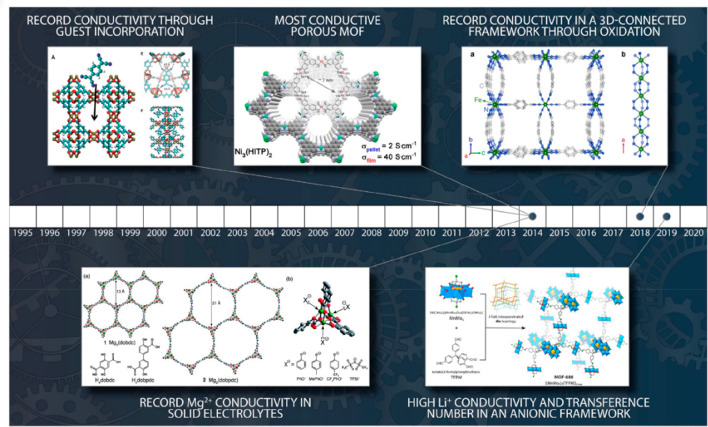
Examples of metal–organic framework hybrid materials (MOFs) with groundbreaking conducting properties (from REF Freund et al. *Angew Chem* 2021). Intrinsically hybrid materials, MOFs have grown into a category on their own and have led to the development of hybrids for which they act as the host framework, even leading to MOF-COF hybrids. Reprinted with permission from ref (85). Copyright 2021 John Wiley & Sons.

Indeed, MOFs and perovskite photovoltaic materials are two relevant examples of intrinsically hybrid materials that grew into their own trees, reinforcing the idea of the hybrid landscape growing from a tree to a meadow. MOFs are the best example of intrinsically hybrid compounds with inorganic moieties being orderly and controllably separated by organic spacers in crystalline phases with controlled porosity and properties.^13,27−30,79−85^ This “reticular chemistry” approach allows a rational design method for creating various metal–organic frameworks and covalent organic framework (COF) structures.^80,85,86^ The main idea of reticular design is to connect organic and inorganic molecular building units with strong directional chemical bonds to form stable crystalline extended structures. A significant advantage of this method is the ability to control the pore size of MOFs and COFs by changing the length of the organic linkers used without changing the framework’s underlying topology. The past decades have seen immense possibilities of reticular design resulting in a wide range of MOF (>100 000) and COF (>570) structures that have unique properties (Figure 5).^85,87^ The great tunability of MOFs and COFs allows the design and synthesis of materials with a huge variety of properties, making MOFs and COFs a great solution for a large variety of applications such as gas storage and separation, vapor sorption, catalysis, biomedical applications, chemical sensing, and electronic and ionic conduction (such as electrode development).

Perovskite photovoltaics is another example of what began as an incipient family of hybrid materials^1,88^ then turned into a whole new realm, with lead perovskites taking the central role and lead-free derivatives trying to circumvent the problems associated with toxic lead and trying to fulfill the safe-by-design guidelines.^89^

Regarding the methods of synthesis of hybrid materials, they are as varied and extensive as the chemistries of their components. Table 1 is an attempt to summarize this extensive palette and represents the strong rooting of hybrid materials in many fields of science and their huge potential to affect a vast variety of applications.

**Table 1:** Summary of the Different Synthetic Methods for Obtaining Hybrid Materials

Method	Description	Advantages	Disadvantages	Applications of interest	ref
Sol–gel	This technique involves the hydrolysis and condensation of precursor molecules, usually metal alkoxides, to form a gel-like material that can be further processed to obtain the desired hybrid structure	• Versatile, and simple	• Time-consuming processing	• Optics	(7, 12−14, 22, 31, 43−54, 57, 90−96)
		• Cost-effective and scalable	• Critical selection of precursors and parameters	• Electronics	
		• Control over the composition, morphology, and structure	• Parameter-sensitive (pH, temperature, solvent composition...)	• Catalysis	
		• Suitability for creating complex structures	• Scalability accessible but challenging	• Energy storage	
		• Enable the synthesis of organic–inorganic composites and functional coatings	• Shrinkage and cracking upon drying	• Sensing	
			• Sensitive to contaminations	• Biomedical engineering	
Self-assembly	This technique relies on the spontaneous organization of molecules or nanoscale building blocks into ordered structures driven by noncovalent interactions	• Versatile	• High influence of reaction parameters that might limit the control over the assembly.	• Drug delivery	(12, 13, 24, 36−42, 54)
		• Allow combining different components with complementary properties.	• Sensitivity to environmental factors. Susceptible contamination and purity issues.	• Biomedical imaging	
		• Well-defined structures and functionalities can be achieved.	• Reproducibility Challenges	• Tissue erngineering	
		• Final hybrid material properties and functionalities can be tailored to address a specific application	• Limited scalability	• Sensors/biosensors	
			• Limited Material Compatibility	• Optoelectronics	
				• Coatings	
				• Energy storage and fuel cells	
Layer-by-layer (LbL) assembly	This technique is based on sequential adsorption of alternating layers of dissimilar materials onto a substrate, resulting in a multilayered material. Each deposition step is followed by rinsing to remove any unbound or loosely attached material.	• Allows precise control over film thickness	• Time-consuming and labor-intensive process	• Coatings	(12−14, 41, 75, 97)
		• Precise control of the composition and functionality	• Limited thickness and scalability	• Sensors	
		• Integration of diverse components with complementary synergetic properties.	• Challenging reproducibility and uniformity between layers	• Drug delivery systems	
		• Multilayer films, coatings, or complex assemblies can be obtained.	• Interlayer diffusion between layers.	• Tissue engineering scaffolds	
			• Delamination issues (weak interlayer adhesion)	• Optoelectronic devices	
			• Incompatibilities between materials. Its proper selection is crucial.	• Piezoelectrics	
In-situ polymerization	This method for the preparation of hybrid materials is based on the simultaneous polymerization of monomers and the formation of inorganic materials, resulting in a covalent interaction between organic and inorganic components.	• Control over the composition	• Difficulties to control the reaction. Need proper optimization of the conditions and concentrations.	• Catalysis	(12, 13, 38, 44, 59, 74, 98−100)
		• Controlled morphology and structure.	• Polydispersity issues	• Energy Storage	
		• Enhanced mechanical properties.	• Compatibility between monomers and precursors on the hybrid material formation.	• Sensing	
		• Versatile: varied materials can be prepared by choosing the correct precursors.	• Reproducibility and scalability issues	• Biomedical Engineering	
Template assisted synthesis	The preparation of the material is conducted by using a template or scaffold that acts as a guide for the formation of the desired nanostructure.	• Precice control of the size, shape, porosity, and composition	• Template removal usually may require hash conditions or time-consuming processes.	• Catalysis	(13, 24, 29, 63, 71, 101−103)
		• Various templates can be employed such as porous materials, biological templates, sacrificial templates, and sefl-assembled nanostructures.	• Limitate to the suitability and availability of the templates.	• Energy storage	
		• Ability to create complex structures such as hierarchical nanostructures.	• Additional post-treatment steps might be required for more precise control.	• Sensors	
			• Low scalability to large production.	• Electronics	
				• Biomedical	
Solvothermal/hydrothermal synthesis	These two methods are based on high temperature and pressure in aqueous (hydrothermal) or organic solvents (solvothermal) to promote the nucleation and growth of the nanomaterials.	• Versatile	• Specialized reaction vessels are required to work with the high pressure and temperatures required.	• Energy storage and fuel-cells	(12, 57, 64, 72, 73, 103−105)
		• Well-defined hybrid structures	• Limited solvent compatibility	• Catalysis	
		• Controlled composition and morphology	• Challenging control over reaction kinetics.	• Environmental remediation	
		• Defined crystallinity	• Formation of undesired phases or byproducts.	• Photovoltaics	
		• Can be combined with other methods	• Post-treatment steps might be needed.	• Optoelectronics	
		• Scaling-up technique		• Sensing	
				• Biomedical	
Microwave-assisted synthesis	Normally related to solvothermal synthesis. It reduces the time to a few minutes and uses a lower temperature.	• Fast	• Specialized equipment is needed. Limited applicability.	• Catalysis	(12, 65)
		• Low energy consumption	• Different microwave absorption and heating selectivity of the materials may affect the homogeneity.	• Energy storage and fuel-cells	
		• Can significantly alter the reaction kinetics compared to conventional heating methods, reducing the energy barrier in the formation of nanomaterials.	• Can induce material degradation.	• Sensing and detectors	
				• Photovoltaics	
				• Environmental remediation	
				• Optoelectronic	
				• Biomedical	
Atomic layer deposition (ALD)	Thin film deposition technique that utilizes self-limiting surface reactions, enabling to control of the growth of thin films with atomic precision.	• High precision and accurate thickness.	• Complex Process and Equipment	• Coatings	(12, 77, 106, 107)
		• Nonsolution-limited method (solubility, temperatures...)	• Limited scale-up potential	• Energy storage	
		• Hybrid thin film deposition by sequentially reacting different precursor gases.	• Limited deposition to solid substrates.	• Optoelectronics	
		• Controlled composition	• Limited material compatibility with the technique	• Microsystems	
			• Slow deposition rates	• Sensors/biosensors	
			• Limited film thickness		
Chemical vapor deposition (CVD)	Based on the decomposition of precursor molecules in the vapor phase to deposit thin films or nanomaterials onto a substrate.	• Synthesis of high-quality nanomaterials	• Limited material compatibility with the technique	• Microelectronics	(12, 13, 107, 108)
		• High variety of nanomaterials can be prepared by choosing the correct precursor gas.	• High-temperature requirement	• Optoelectronics	
		• Precise control over composition and structure	• Limited uniformity and controlled deposition for large-area substrates	• Gas sensors	
		• Accurate thickness and crystallinity	• Limited to planar substrates.	• Catalysis	
			• Equipment Complexity and Cost.	• Biomedical	
				• Coatings/barriers	
				• Energy storage	
				• Flexible electronics	
Chemical bath deposition	A solution-based technique used to deposit thin films or nanoparticles onto a substrate, like CVD, but it involves the reaction of precursor solutions in a chemical bath under controlled conditions.	• Simple and low cost	• Slow deposition rate	• Optics	(13, 108, 109)
		• By manipulating the bath composition and reaction parameters different hybrid materials with desired compositions and structures can be prepared.	• Lack of precise control. Temperature, pH, and concentrations significantly influence the deposition rate and quality.	• Sensors	
		• Ability to coat complex-shaped substrates	• Sensitive to contaminants	• Energy storage and harvesting.	
		• Controlled thickness and composition	• Limited for specific materials	• Coatings/barriers	
			• Low crystallinity		
			• Postdeposition treatments might be needed.		
Electrochemical deposition/electrodeposition	This technique uses an electric current to deposit metals or other materials onto a conductive substrate from an electrolytic solution.	• Versatility	• Limited material compatibility	• Coatings	(13, 29, 42)
		• Deposition of different materials such as metals, oxides, polymers, or organic molecules (grafting)	• Electrode substrate dependency. The substrate can affect the final properties.	• Optoelectronics	
		• Control over the deposition process and rate	• Limited control over the thickness and uniformity	• Electrocatalysis	
		• Tailored properties	• Prone to pore formation cracks, or dendritic structures in the deposited films	• Energy storage	
			• Scale-up challenges.		
Coprecipitation method	In this method, hybrid nanomaterials are prepared by simultaneous precipitation of multiple components from a solution or a mixture of precursors.	• Simplicity	• Lack of Control over Particle Size and Morphology.	• Catalysis	(12, 110−113)
		• Cost-effective	• Limited control over composition; heterogeneity issues	• Sensors	
		• Intimate combination of the hybrid material precursors.	• Formation of agglomerates	• Drug delivery	
		• Enhanced properties in the final hybrid structure.	• Prone to form impurities	• Environmental remediation	
Sonochemical synthesis	This method uses high-frequency sound waves (typically above 20 kHz) in a liquid medium containing precursor materials. The intense acoustic cavitation created by the ultrasound waves generates localized high temperatures and pressures, resulting in various physical and chemical effects that facilitate the synthesis of hybrid materials.	• Acceleration of the synthesis	• Limited scalability	• Catalysis	(14, 114, 115)
		• Homogeneous distribution of the different components	• Parameter sensitivity	• Functional coatings	
		• Fine particle size control	• Formation of undesired byproducts	• Sensing	
		• Morphology is controlled by adjusting the reaction conditions.	• Potential material degradation of highly sensitive precursors	• Biomedical applications	
		• Reduced temperature synthesis (compared with other methods)		• Energy storage	
		• Versatile: different combinations can be used to produce hybrid materials (such as nanoparticles, polymers, carbon materials, and biomolecules...)			
Electrospinning/electrospray	Electrospinning and electrospray techniques use an electric field to produce polymeric fibers (electrospinning) or particles (electrospray) with diameters and sizes in the nanometer range, deposited onto a conductive substrate.	• Tuneability and versatility	• High dependence on the environmental conditions (temperature and humidity) to control the fiber or particle morphology.	• Tissue engineering	(12, 13, 57, 116−118)
		• Allow the incorporation into the polymer matrix of other nanomaterials such as metallic and ceramic nanoparticles, nanocarbon, and/or MOFs	• Optimal parameter dependence	• Environmental remediation	
		• Scaling-up technique	• Prone to aggregation and to limited production rate for electrospray	• Energy storage	
			• Postprocessing may be required.	• Drug delivery	
				• Sensors	
Spray pyrolysis	This method involves the atomization of precursor solutions into fine droplets and the subsequent thermal decomposition of the droplets to form solid particles or thin films. This technique allows for the synthesis of hybrid materials by incorporating multiple precursors in the spray solution	• Controlled composition and morphology adjusting the process parameters (temperature, flow...)	• High influence of process parameters	• Catalysis	(12, 14, 119, 120)
		• Simplicity	• Low control over particle size	• Energy storage	
		• Versatile	• Aggregation and coalescence issues	• Thin films fabrication	
		• Wide range of hybrid materials can be produced.	• Challenging scale-up	• Sensors	
			• Energy consumption		
			• Additional post-treatment steps might be required.		
Mechano-chemical synthesis	Also known as ball milling, it involves the use of mechanical force to induce chemical reactions and synthesis of nanomaterials.	• Simple and low-cost	• Time-consuming process	• Catalysis	(12, 64, 121)
		• Scalable	• Slow reaction rates	• Energy Storage	
		• High yield	• Limited scope of reactions	• Environmental remediation	
		• Solvent-free processing	• Impurities might be obtained from the jars and grinding balls.	• Pharmaceutical/Drug delivery	
		• Control over the composition	• Broad particle size distribution		
		• Can be also liquid or ultrasonic-assisted.	• Additional post-treatment steps		
Microemulsion and reverse micelle techniques	This technique involves surfactants and organic solvents to form stable nanoscale droplets or micelles in a continuous phase, allowing the formation of different nanomaterials in the confined space inside the droplets.	Simple and easy	• Limited control over particle size distribution and morphology	• Drug delivery	(13, 44, 93, 95)
		A wide range of materials can be used from hydrophobic to hydrophilic, including organic and inorganic compounds, polymers, and biomolecules.	• Limited material compatibility	• Cosmetics	
		Controlled particle size and morphology	• Limited loading capacity	• Biomedical applications	
		Unique microenvironments can be created in confined spaces.	• Affected by diffusion rates	• Catalysis	
		High homogeneity and stability.	• Post-treatment needed		
		Controlled release and delivery.	• Environmental impact by surfactants		

## 3 Structure–Property Relationships

Composition is obviously important in the design of hybrid materials, but the structure is equally crucial. Indeed, how structure determines properties is, in itself, a fascinating topic. From molecular chemistry to materials science, all hierarchical levels of structure have paramount importance in determining physical and chemical properties. Hybrid materials are no exception, of course. Furthermore, their multimaterial nature provides opportunities for the design of sophisticated nanostructures and molecular architectures leading to the fine control of their properties. In this section, we have selected a few case studies concerning different structures for a variety of properties and applications.

**Figure 6:**
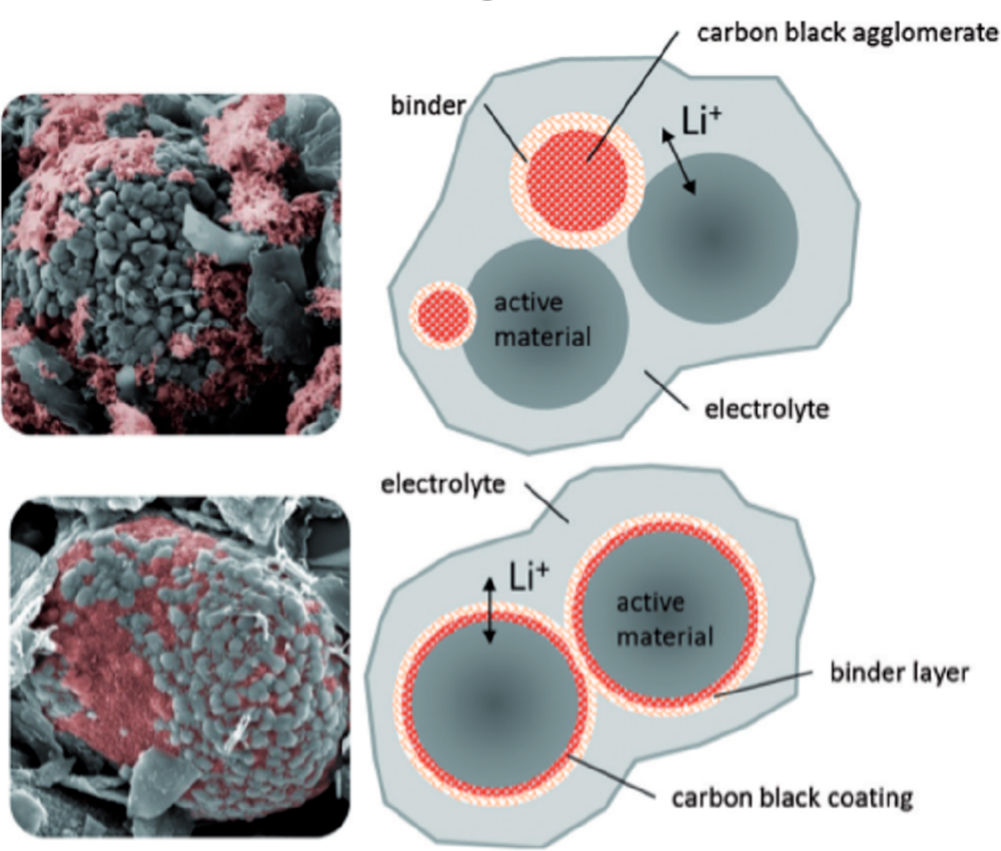
An example of the importance of microstructure in the field of hybrid nanocomposite electrodes: The top images show a real SEM photograph and a schematic rendering of the improper agglomeration of carbon and binder. The bottom images represent the corresponding SEM and diagram of a sample electrode with a proper coating of the active material, resulting in improved performance. Reprinted with permission from ref (123). Copyright 2015 John Wiley & Sons.

The electrochemical properties of electrodes from supercapacitors and batteries are a fitting example of a hybrid material in which the structure is a key factor. Current electrodes usually are composed of the active materials (which store energy), a conductive component, and a binder. How they are combined has evolved from the empirical simple mixture in the past to more sophisticated approaches that take into account the critical role that hierarchical structures generated in the electrode have on the electrode performance.^34,122^ Most active materials are insulators or have low electrical conductivity to work as electrodes on their own. That is why a conductive component is needed, which must generate a percolation network allowing an efficient transport of electrons from the current collector to all of the particles of active material. However, the combined structures of each of the components should be optimized. Figure 6 illustrates the importance of a properly structured composite. Thus, the top images show a real SEM image and a schematic drawing of a suboptimal combination of the three elements (active conducting and binding components). On the other hand, the bottom images in Figure 6 show electroactive particles properly coated with conducting carbon and the binder uniformly distributed but without blocking percolation pathways, which correspond to an optimized, properly working electrode.^123^

**Figure 7:**
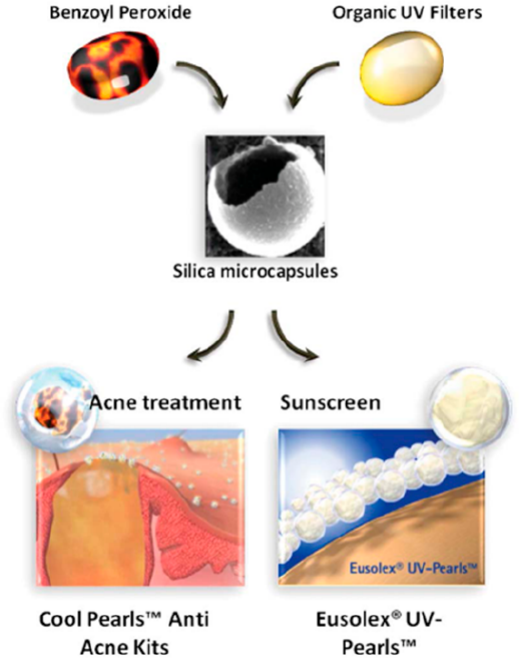
Encapsulation as a primary structuration method in hybrids. From drugs for acne treatment to UV-filters for sunscreens, silica microcapsules provide optimal delivery. Reprinted with permission from ref (12). Copyright 2009 Royal Society of Chemistry.

Encapsulation is another example of a hybrid material in which the structure is a key feature. Some applications use hybridized inorganic nanoparticles as active pharmaceutical ingredients, vectors, or enablers. Others employ them for in vitro diagnosis in lateral flow devices (immunochromatography) or in delivery systems (drug delivery, implantable biomaterials, and vaccine adjuvants). The field of cosmetics and controlled release of “active ingredients” is the target of these new hybrid compounds; they are especially useful for skin care and protection applications As an example, silica microcapsules can reduce skin contact with harmful organic UV filters by their encapsulation in microcapsules, which is important because these chemicals can produce free radicals that damage DNA. These “UV-pearls” can be mixed with a suitable cosmetic vehicle to achieve high sun protection factors (SPFs), while improving the safety profile as the penetration of the UV filters is significantly reduced. Some companies have already used these “UV-pearls” for sunscreens and daily wear cosmetics (Figure 7).^12^

Structure is also key in biomedical applications in general, polymer bioconjugates, and the conjugation of biomolecules with synthetic polymers is a smart solution to get a hybrid material with advanced properties. In this field, the influence of polymer topology on the properties and applications of polymer bioconjugates is significant. Polymer bioconjugates are hybrid biomacromolecules that combine synthetic polymers with various biomolecules. Branched polymers, such as brushlike, hyperbranched, and dendritic polymers, have been widely used for biomedical applications due to their unique features compared to linear polymers. Therefore, the synthesis of branched polymer bioconjugates has become a promising research area to obtain biohybrid materials with enhanced stability and prolonged circulation times in vivo.

The design of polymer bioconjugates depends on a range of factors, such as the site-specific conjugation chemistry; the size, distribution, topology, and function of polymers; and the architecture of bioconjugates. These factors affect the higher-ordered assemblies and hierarchical structures of polymer bioconjugates in solution, in bulk, and on surfaces. The synthetic tools and methods for creating polymer bioconjugates have been rapidly developed and improved in recent years. Moreover, the understanding of biomolecule structure and function has also been deepened, leading to novel constructs and applications in materials science.

The field of polymer bioconjugates is constantly evolving and expanding. The synthetic chemistry of macromolecules offers a wide range of possibilities that surpass those found in nature. However, nature also provides inspiration and guidance for creating complex and functional systems that can communicate with and regulate themselves. The future of polymer bioconjugates may lie in establishing the molecular principles of how these macromolecules can be customized and integrated into artificial environments. The potential applications of these biohybrid materials are enormous and diverse.^60,124−126^

**Figure 8:**
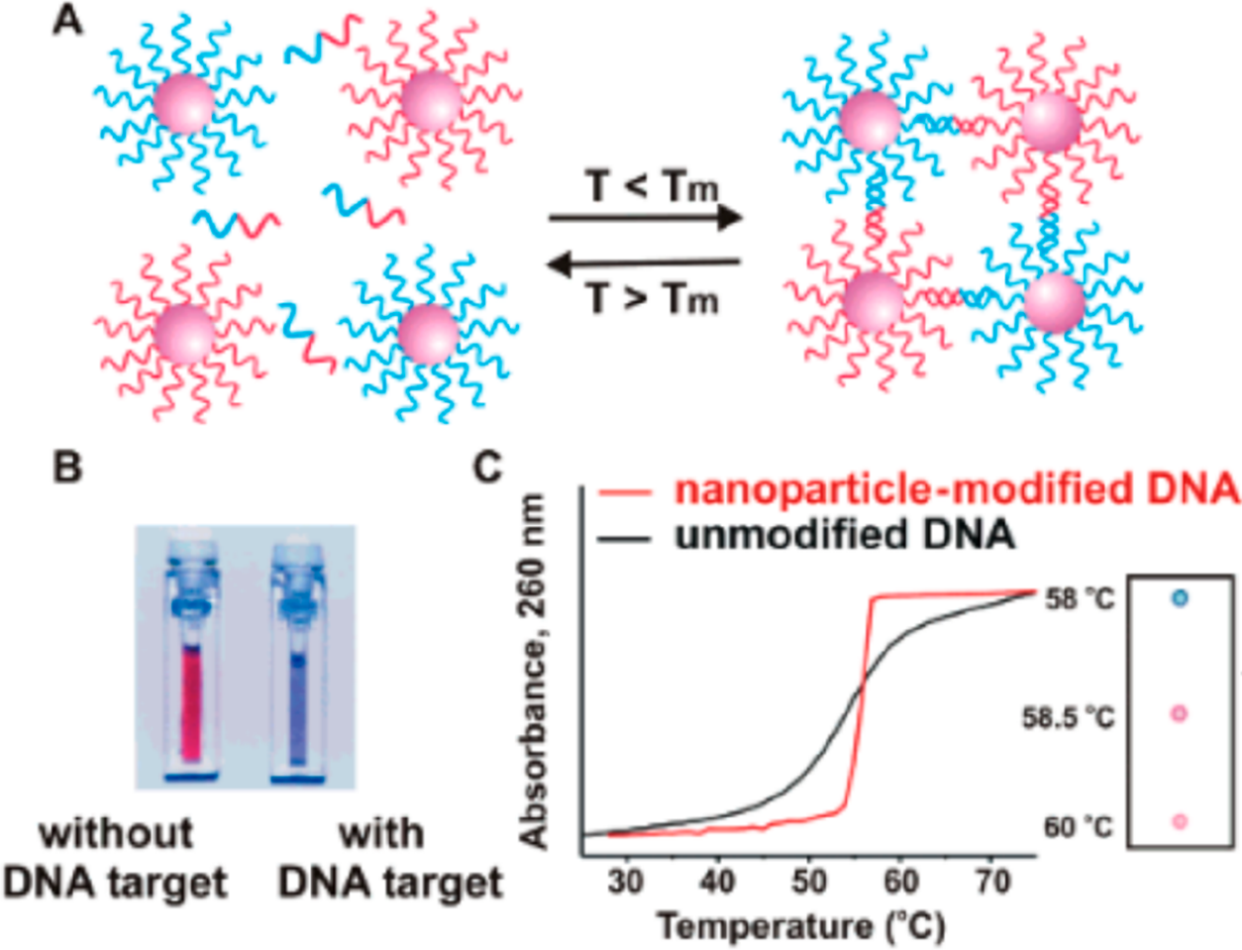
Oligonucleotide-functionalized gold nanoparticles aggregate in the presence of complementary target DNA (A), resulting in a color change from red to blue (B), which can be monitored by UV–vis spectroscopy (C). Reprinted with permission from ref (127). Copyright 2005 American Chemical Society.

Finally, biodetection applications also require a tailor-made adjustment of the physical, chemical, and structural properties of nanomaterials. For example, metal nanoparticles and quantum dots can be tuned to have different optical properties, such as emission, absorption, and scattering, by changing their size, shape, and composition.^53,94^ This allows for the detection of multiple analytes simultaneously using different colors of light; nanowires and nanotubes can also be modified to have different electrical properties, such as conductivity and resistance, depending on the presence of target analytes. Furthermore, nanomaterials can be functionalized with biomolecules or small molecules to enhance their specificity and affinity for various targets. These advances in nanotechnology enable the fabrication of nanoscale arrays of sensors on surfaces. One example of such a sensor is the colorimetric assay based on gold nanoparticles that change color when they aggregate in response to DNA hybridization. The aggregation of gold nanoparticles alters their surface plasmon resonance and scattering properties, resulting in a visible color change from red to blue. This assay can be used as a simple and rapid test for nucleic acid detection by spotting the solution onto a white support (Figure 8).^127^

## 4 Applications

The variety of compositions and chemical structures that arise from all the possible combinations of organic and inorganic components makes hybrid materials not only suitable but also an excellent option for a wide range of applications. On top of that, the synthesis method can be tailored to achieve specific properties, providing a powerful tool to address current technological problems.^20^ It is evident that the applications of hybrid materials are numerous and diverse, spreading through many fields, including energy storage, catalysis, sensing, photonics, and biomedicine. However, within each of these fields, the applications of hybrid materials are vast, and describing them all in one review has become an impossible task. Again, it is inevitable to think about the tree metaphor (Figure 1), in this case a branching tree, focusing now on the different main branches and individual leaves, each with its numerous nerves. Thankfully, it is common now to find reviews dealing with hybrid materials that find application in only one specific area, and in this section, we will provide the readers with a summary of these articles attempting to assist them in their journey through the world of hybrid materials and their applications. In this regard, those looking for a first glance at the properties and fields of application, should refer in a first instance to general reviews^3,12,20,95,128^ which usually include a brief description of possible uses of hybrid materials or current technological advances. Based on these reviews, the myriad of applications found for this kind of material have been grouped as follows:

### 4.1 Optics and Photonics

This is one of the first fields in which hybrid materials found application. By doping inorganic materials like glass, clays, silica, and zirconia with organic dyes, it has been possible to improve the photostability of the chromophore compound, an approach trying to reproduce one of the oldest hybrid materials, Maya blue. Since these first studies, more sophisticated developments seeking to achieve different optical properties have been developed. For example, polymer-based hybrids containing thin films of Ti or Zr metal oxides, show refractive indexes higher than those of the individual components.^3^ Furthermore, fast and reversible photochromic materials can be obtained by limiting the organic–inorganic phase interactions by simply modifying the dye-doping procedure.^53^ The list of optical devices that can be produced with hybrid materials is, as expected, quite large, but some examples include high or low refractive-index materials, waveguide materials, photochromic^94^ and electrochromic materials, nonlinear optical materials,^53^ photodetectors, and decorative coatings. The reader interested in this particular topic is encouraged to consult the thorough review by Lebeau and Innocenzi,^53^ where a comprehensive list of the applications and references can be found. It is also worth mentioning that this review is mainly focused on sol–gel materials. However, it does provide a good idea of the potential of hybrid materials in the fields of optics and photonics.

### 4.2 Biomedical Applications

**Figure 9:**
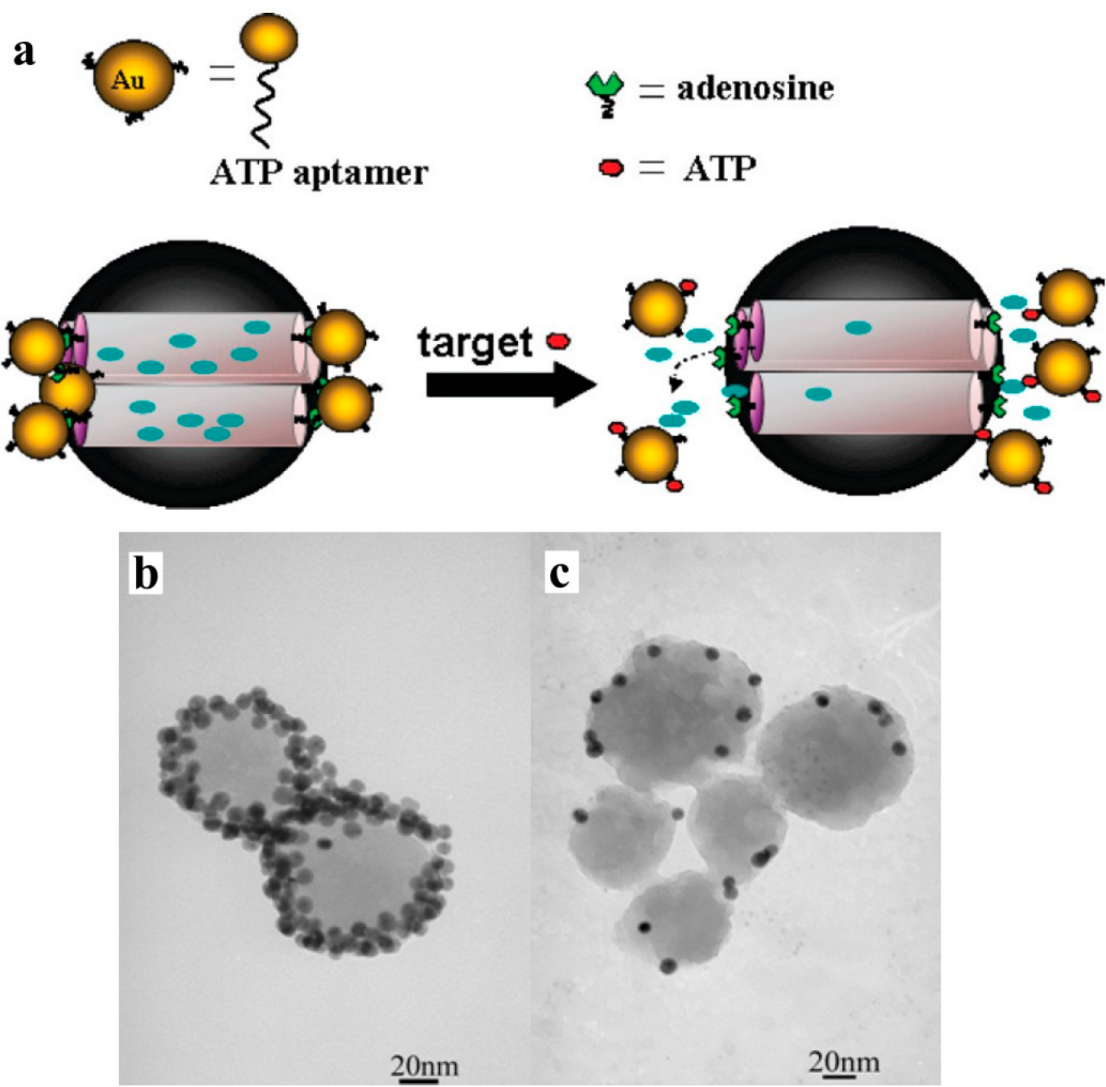
(a) Scheme showing working principle of Au nanoparticle capped-mesoporous silica as responsive controlled drug delivery systems. TEM images of these particles in the (b) before and (c) after being exposed to ATP rich environment. Adapted with permission from ref (131). Copyright 2011 American Chemical Society

The versatility of hybrid materials makes them suitable for several applications within this field, like tissue engineering, drug delivery, dentistry, and bioimaging. Once more the idea is to combine the properties of organic and inorganic phases, keeping in mind that the latter in this case must be nontoxic and biocompatible. This is one of the reasons why silica-based hybrids are currently the most popular ones, especially since it has been approved by the FDA for human trials.^66^ Silica nanoparticles provide a stable platform which can be easily functionalized with different materials, biomolecules, and targeting ligands, thanks to the presence of Si–OH groups on its surface. The interaction between silica and the organic moieties can be either weak (drug delivery) or strong (tissue engineering) depending on the requirements of the materials.^129^ Moreover, porous structured silica particles (MCM-41 or SBA-15 type)^54^ can be used as carriers of bioactive molecules entrapped within the pores by “gatekeeper” molecules, polymers, or even metal/metal oxide nanoparticles. These are usually designed to respond to either external triggers such as temperature, light, magnetic field, ultrasound, and electricity or internal triggers such as glucose, enzymatic activity, pH, ATP, and glutathione (Figure 9), which makes it possible to target only affected areas. Even more complex silica-based materials, containing both therapeutic and imaging agents,^66,128^ have been devised for the real-time monitoring of drug release and therapeutic response (theragnostic systems). Silica-based 3D scaffolds have also been proposed for bone tissue engineering since it is possible to graft osteoinductive agents which act as attracting signals for bone cells promoting tissue growth and regeneration. Other types of materials that are attracting interest in this field are metal–organic frameworks (MOFs)^79,128^ which can be easily combined with biopolymers to comply with biocompatibility requirements.^130^

### 4.3 Catalysis and Electrocatalysis

**Figure 10:**
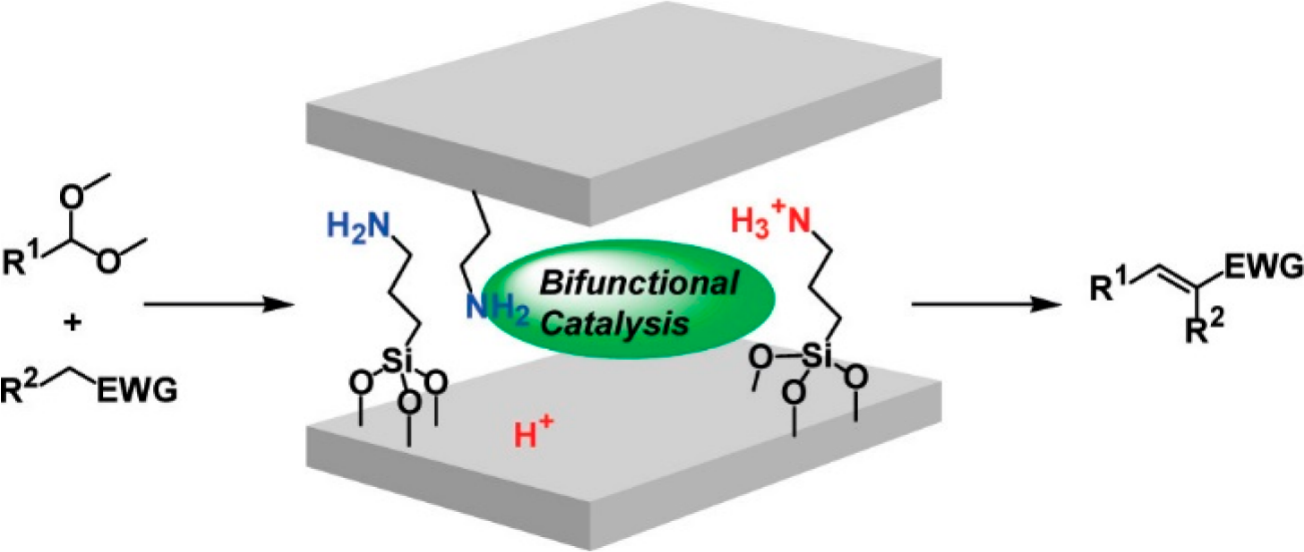
Acidic montmorillonite-immobilized primary amines as acid–base bifunctional catalysts for cascade reaction. Reprinted with permission from ref (15). Copyright 2009 American Chemical Society.

Hybrid materials were first used in catalysis to increase the stability of organic or organometallic homogeneous catalysts. In this type of material, the organic phase was retained within the structure of the inorganic phase only by weak interactions (van der Waals, hydrogen bonds, or electrostatic), falling within the class I classification proposed by Sánchez and Ribot.^33^ Even though leaching, pore blocking, and subsequent deactivation of the catalyst are still major issues of these hybrids, this approach solves the most important problems of homogeneous catalysis such as product separation or recovery and catalyst reusing. Organic molecules occluded within the cavities of zeolites are one example of catalysts that have been prepared in this way. A second stage in the development of hybrid catalysts was mainly focused on overcoming the main drawbacks of Class I type materials by establishing a covalent bond between both phases (Class II). This ensures not only that active organic molecules are stable (no leaching) but also that they are homogeneously distributed so the whole material (pores and cavities) can be fully exploited. The latter is also true since the organic counterpart is now confined within the walls of the solid and does not block the internal channels of the solid, allowing reactants to diffuse inside. Silica and silica–alumina have been frequently employed as inorganic supports due to their large specific area and high number of reactive sites, which can be used to anchor the organic compounds. However, layered oxides have also been used to produce bifunctional acid–base catalysts (Figure 10). A final comment must be made about a third kind of hybrid materials that has gained popularity in this field, namely, organic modified/functionalized metal and metal oxide nanoparticles.^132,133^ This is an emerging area that uses surface functionalization to manipulate the catalytic properties of nanoparticles. As an example, it has been reported that modification of Ru particles with ethylenediaminetetraacetic acid (EDTA) changes the selectivity of the catalyst in Fischer–Tropsch synthesis.^134^

### 4.4 Energy Storage and Conversion

In the field of energy storage, hybrid materials have attracted a lot of attention since combination, for example, of carbon materials with pseudocapacitive materials (metal transition oxides or conductive polymers) can help overcome the limitations they show individually and boost the performance of supercapacitors.^17^ Going a step further, faradaic materials could be incorporated in the network of capacitive-like materials to prepare electrodes with a hybrid electrochemical response. The latter provides a way to close the gap between batteries and supercapacitors in terms of the energy and power density. For energy storage, the structure of the materials (see the previous section) plays a fundamental role in the performance of the resulting devices. For example, decreasing the size up to the nanoscale can even lead to capacitive-like behavior of materials commonly known as “faradaic”. The possible combinations are infinite, and even multiple hybridizations (carbon/metal oxide/conductive polymer) have been considered.^135^ On this topic the reader can find specific reviews such as those by Gómez-Romero et al.^69,136^ dealing with polymer–metal oxides (including polyoxometalates) hybrids or that of Reddy et al. which focuses mainly on CNT-based hybrids^71^ for energy storage applications. Solar energy harvesting is another field that is taking advantage of the benefits of hybridization. Organic–inorganic perovskites are materials with the typical chemical formula ABX_3_, where B is a divalent cation and X a halogen anion, but unlike traditional perovskites A is an organic cation (e.g., methylammonium).^137^ These materials have gained popularity due to their ease of preparation, low cost, and high efficiency. Also, the right combination of metal and organic cations as well as halogen anion can provide the desired bandgap.^138^ Although these materials show great potential, commercialization is just starting and other aspects need to be addressed.

### 4.5 Sensing

**Figure 11:**
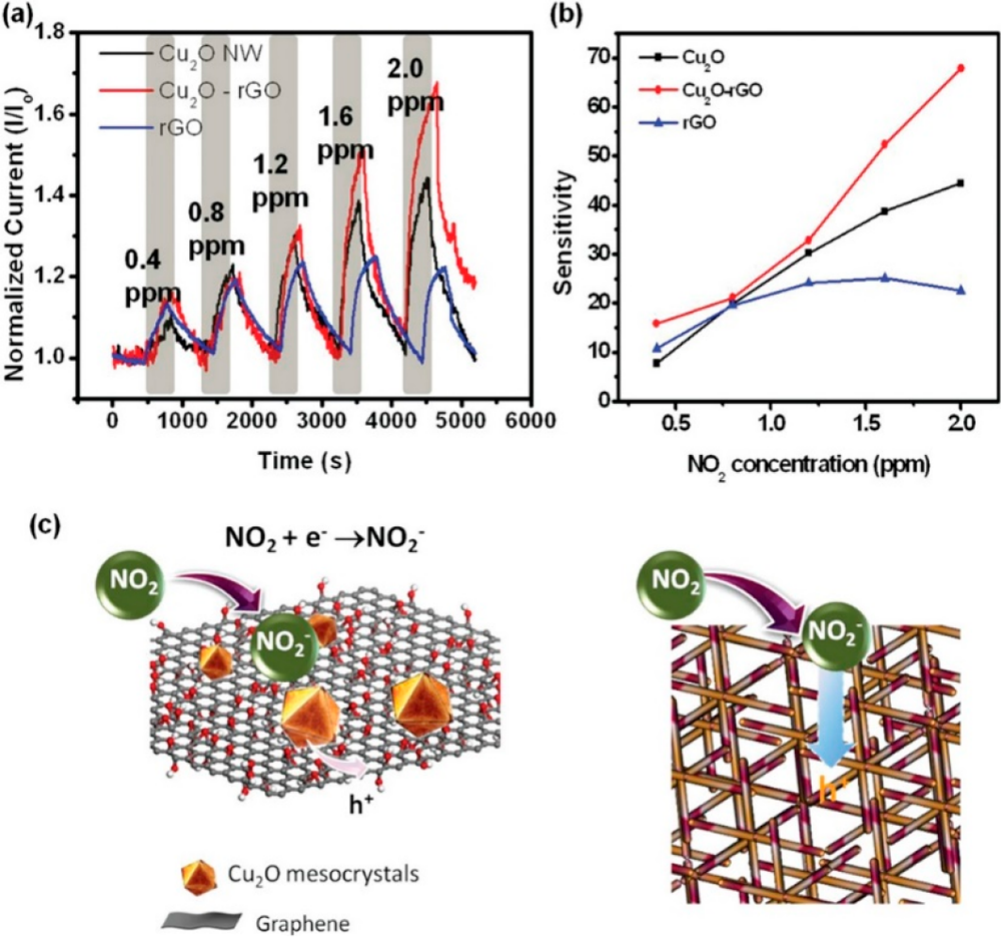
(a) Dynamic response of Cu_2_O nanowires, rGO-Cu_2_O, and rGO materials under increasing NO_2_ concentrations, showing the synergistic effect of both phases. (b) Sensitivities of NO_2_ sensors for the three devices. (c) Mechanism of NO_2_ sensing of rGO-Cu_2_O. Reprinted with permission from ref (32). Copyright 2012 American Chemical Society.

The field of sensors is overly broad, since they can be used to detect gases, chemical species, biomarkers in biologic systems, humidity, mechanical deformations (strain or pressure), temperature, or UV-radiation. This in turn means that the composition of hybrid materials developed for sensing applications can be diverse. Thus, for those interested in this topic, the review by Wang et al.^139^ is a good starting point, where preparation methods, sensing configuration, shape of sensor, and an overview with examples of materials used in diverse kinds of sensors is provided. According to these authors, the most general approach consists of protecting the organic sensing molecules within the inorganic matrix. However, as observed for other applications, hybrids can be designed in such a way that there is a synergistic effect between both components, enhancing the performance when compared to individual materials (Figure 11). Carbon-based hybrids, e.g., graphene^140^ and CNT,^141^ have been extensively considered for this purpose to take advantage of the high conductivity, specific area, and good thermal stability. Besides, the surface of these nanocarbons is already reactive, so they can act as sensors. In this case, hybridization with either polymers or metal/metal oxide nanoparticles can further enhance their sensitivity. Photofunctional hybrid sensors have been prepared by encapsulation of metal ions or nanoparticles within the structure of MOF. These materials have proved to be efficient in the luminescent detection of a wide range of biomarkers.^79^ Another interesting review in this field deals with the combination of phthalocyanines and metal nanoparticles to obtain hybrid materials with excellent properties for chemiresistive and electrochemical sensing.^142^ Finally, it is worth making a short comment on the development of flexible sensors. This emerging field has many interesting applications, such as electronic skin, medical electronics, environmental detection, and wearable devices, which could benefit from the properties of hybrid materials.^143^ For example, it is not hard to imagine how the combination of flexible but low-sensitivity organic materials with inorganic semiconductors can accelerate the implementation of these devices in real-life applications. An excellent summary of the possibilities and current limitations of these devices has been provided by Ren et al.,^143^ together with over 100 references for interested readers.

### 4.6 Electronics

The term “electronics” is quite broad, and many different applications fall within this topic, including photovoltaics, which in this paper have been discussed briefly as energy conversion materials. However, in several reviews, hybrid materials for solar cells are included in the electronic applications section, or to be more precise under the optoelectronic label. Although both classifications are correct, here we focus on other applications such as transistors, diodes, and memory devices. The reader should keep this in mind while consulting the articles cited in this section since they do not strictly follow our approach. Thus, the general review by Mir et al.^128^ includes a brief introduction to the many advantages of hybrids for electronics (photovoltaics included), electrical memory materials, and flexible devices. This field is mostly dominated by two distinct types of materials: (a) conductive polymer-based hybrids with nanocrystals or nanoparticles of inorganic semiconductors or metal nanoparticles and (b) hybrid halide perovskites. Regarding the first group, the article by Reiss et al.^58^ provides a complete list of interesting materials, the hybridization routes, the electronic properties of the resulting hybrids, and even the characterization techniques used to determine their physicochemical properties. In a similar way, Holder et al.^144^ discussed the application of these materials in the field of optoelectronics, with a special emphasis on light-emitting diodes. The fundamentals aspects and synthesis methods of hybrid perovskites for several applications (transistors, memory devices, and artificial synapsis) are properly discussed by Choi et al.,^145^ providing a great starting point to understand how these materials could revolutionize the field. Finally, we would like to mention the work by Hwang and Lee^89^ which deals only with the design of memory devices based on hybrid materials.

### 4.7 Environmental Remediation

**Figure 12:**
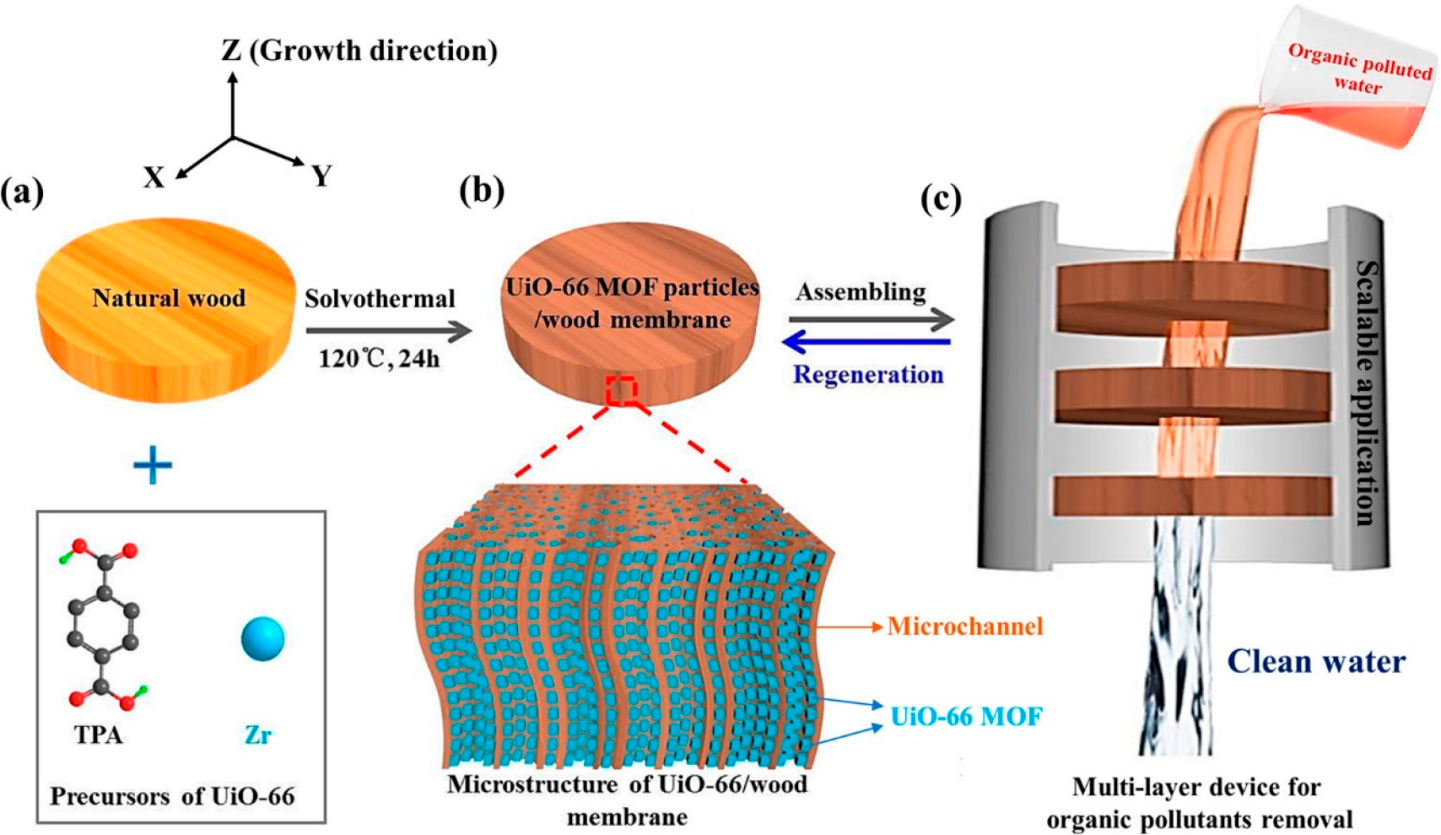
(a) Scheme of the in situ synthesis process and (b) of the resulting structure of a UiO-66 MOFs-wood hybrid membrane for efficient organic pollutant removal. (c) Filter built with 3 membranes for large-scale operation. Reprinted with permission from ref (104). Copyright 2019 American Chemical Society.

The design concept of hybrid materials for the removal of hazardous pollutants spans from simple functionalized porous silica materials^63^ to complex polymer–inorganic semiconductor photocatalysts.^146^ Of course, the mechanisms through which these toxic chemicals are eliminated from water courses, industrial effluents, or the atmosphere (in the case of gases) are completely different. Nonetheless, the idea is always the same, i.e., to get the best of both worlds. For example, as has been discussed in previous paragraphs, porous silica materials provide a high surface area platform to which organic molecules can be easily attached. The latter can be chosen to interact with heavy metal ions or organic dyes, which will be adsorbed and fixed at the surface of the silica particles. In this way, by simply recovering the solid particles, remediation of aqueous media can be efficiently done. Using a similar concept, researchers have been working on the development of magnetic hybrid sorbents which can then be separated from the treated sample by means of a magnetic field.^147^ Graphene, carbon nanotubes, and MOF based hybrids with iron or iron oxide as magnetic phase have been studied, among others. Three-dimensional graphene hybrid materials with nanoporous and microporous structures have been produced for water purification and environmental monitoring. These materials have been tested as filtration membranes, adsorbents, and as pollutant degradation agents.^148^ Other promising devices in this field are MOF-based hybrid filters (Figure 12). With high flux and low-pressure drop, they show great potential for both air and water purification.^149^

### 4.8 Coatings

The use of hybrid materials as protection coatings, especially for corrosion inhibition, is one of the most obvious and straightforward applications. In a sense, these materials are the evolution of traditional paints, where particles of inorganic oxides are dispersed in a polymeric matrix. With this in mind, a lot of effort has been put into the design and preparation, mostly through sol–gel methods, of polysiloxanes coatings. One of the advantages of this kind of material is the capacity to react with the −OH groups at the surface of metallic surfaces, leading to a strong substrate–coating interaction (i.e., great adhesion).^150^ Class II hybrids can be tuned to have high hydrophobicity, good corrosion protection (barrier effect), low dielectric constants, or good scratch resistance, fulfilling all the requirements of a corrosion mitigation material.^51^ However, the possibility of hosting different functionalities within the siloxane matrix widens the range of applications for hybrid materials. Self-healing, self-cleaning, antifouling, fire-retardant, and antireflective coatings have already been prepared.^151^ Regarding the suggested literature, the mini-review by Zvonkina and Soucek^151^ introduces the topic, giving some details on the different types, preparation, and applications of sol–gel hybrid materials as coatings. Moving on, in-depth information on the use of hybrid coatings for corrosion protection (and microbiologically induced corrosion) can be found in the articles by Figueira et al.^50,51^ and Al-Saadi and Raman.^150^ Finally, the review by Zhang et al.^96^ summarizes the advances and design strategies of polymer–ceramic hybrid antifouling coatings based on chemical hybridization.

### 4.9 Other Applications

At the beginning of this section, the multiplicity of hybrid materials was highlighted, mentioning how the latter creates an entire world of opportunities when it comes to applications. The most common ones have been discussed briefly in previous paragraphs, but it should be kept in mind that this list is surely incomplete. Being a fast-growing field, new materials are constantly being prepared, which may find application in areas that have not been considered before. While going through the literature we have come across mentions of hybrid materials being used in aerospace and automotive applications^152^ or as membranes for forward osmosis.^74^ Moreover, biopolymer-based hybrids are currently being studied as functional food-packaging materials.^124^ As a closing remark, we would like to remind readers that this is only a glimpse of the world of hybrid materials and that their applications right now are limited only by our imagination.

## 5 Concluding Remarks: Perspective and Prospective

The classical approach to chemistry is one in which purity is a cornerstone: extraction, purification of mixtures, isolation of compounds, and only then analysis and understanding. But when it comes to the chemistry of materials, purity is not necessarily an asset. On the contrary, complexity frequently reigns even in simple single-compound materials, and of course, in hybrid materials complexity comes standard. Nevertheless, complexity is not a problem but an opportunity for the design and development of new materials with the desired properties, and all of the materials and types of materials that we have discussed in this metareview are good examples of this.

Indeed, in this article we have tried to provide a broad perspective of the expanding world of hybrid materials, from its origins to the development of new hybrid trees of knowledge, trying also to go beyond the mere enumeration of types or applications. Thus, we have strived to underscore common factors that could inspire young researchers to tackle the challenge of hybrid diversity. This approach renounces necessarily going deep into any specific field, but this is not a problem in view of the growing number of diverging topics and reviews dealing with hybrid materials. The practitioner searching for specific and specialized knowledge will always find excellent reviews, from hybrids for energy storage to theragnostics. This metareview, in contrast, is devoted to a more general and transversal view of a field that is not a single field anymore and tries to convey the awesome feeling that comes when we realize how the chain-reaction development of a topic like that of hybrid materials has led in a few decades to a cascade of new topics and subtopics, even new fields contributing to the development of new materials and new devices for the improvement of our way of life.

A prospective analysis of the development of hybrid materials could include consideration of new design tools, new types of hybrids, and their future impacts.

New design tools should come from both synthesis and analysis. In the synthetic corner, an empirical trial-and-error conventional approach should give way to a more rational approach based on or at least supported by theoretical calculations and modeling.^153^ On the analytical side, the characterization of materials has already grown into a very sophisticated field, with the instruments of nanocharacterization and the power of large installations like synchrotrons put to the service of ever more complex materials like our hybrids.

Future impacts will concern new fundamental knowledge made possible, in part, by those new tools discussed above. New knowledge, driven as always by intellectual audacity, will keep growing and contribute to the explosion of open scientific publications. But hybrid materials will certainly also have a growing impact concerning final, real-world applications. Even as the world experiences contractions of demand due to pandemics and faces a supply crisis, there is still a growing technological market, demanding more and more materials with multifunctional and tailor-made properties, creating the perfect context for hybird materials to thrive.

Finally, concerning the types of materials that are foreseen on the horizon of hybrid materials, there are reasonable predictions, some related to their nature and some to their applications. Concerning applications, it is most likely that hybrids will get consolidated for biomedical applications and that their use will grow in energy-related applications. Concerning the nature of future hybrid materials, it is only natural that their variety will keep growing. This, together with a trend toward growing complexity of multimaterial designs, can be foreseen as a response to the growing trend in tailor-made applications. But in the field of hybrid materials, it is truer than ever that the limits in material design are constrained just by our limited imagination.
